# Early recognition of heart failure in patients with diabetes type 2 in primary care. A prospective diagnostic efficiency study. (UHFO-DM2)

**DOI:** 10.1186/1471-2458-9-479

**Published:** 2009-12-21

**Authors:** Leandra JM Boonman-de Winter, Frans H Rutten, Maarten J Cramer, Anho H Liem, Marcel J Landman, Henk F van Stel, G Ardine de Wit, Guy EHM Rutten, Paulien AW van Hessen, Arno W Hoes

**Affiliations:** 1 Center for Diagnostic Support in Primary Care (SHL), Department of Scientific and Contract Research (WECOR), Bredaseweg 165, 4872 LA Etten-Leur, the Netherlands; 2 Julius Center for Health Sciences and Primary Care, University Medical Center Utrecht, the Netherlands; 3 Department of Cardiology, Heart-Lung Center, University Medical center Utrecht, the Netherlands; 4 Department of Cardiology, Oosterscheldeziekenhuizen, Goes, the Netherlands; 5 Department of Cardiology, Meander Medisch Centrum, Amersfoort, the Netherlands

## Abstract

**Background:**

We hypothesize that the prevalence of unknown heart failure in diabetic patients aged 60 years and over is relatively high (15% or more) and that a cost-effective strategy can be developed to detect heart failure in these patients. The strategy is expected to include some signs and symptoms (such as dyspnoea, orthopnoea, pulmonary crepitations and laterally displaced apical beat), natriuretic peptide measurements (Amino-terminal B-type natriuretic peptide) and possibly electrocardiography. In a subset of patients straightforward echocardiography may show to be cost-effective. With information from our study the detection of previously unknown heart failure in diabetic patients could be improved and enable the physician to initiate beneficial morbidity and mortality reducing heart failure treatment more timely.

**Primary objectives:**

- To assess the prevalence of (previously unrecognised) heart failure in primary care patients with diabetes type 2.

- To establish the most cost-effective diagnostic strategy to detect unrecognised heart failure in these patients.

**Secondary objectives:**

- To assess the impact of heart failure, and the combination of a new diagnosis with accordingly treatment in patients with diabetes type 2 on health status.

**Methods/Design:**

Design: A prospective diagnostic efficiency study.

Patient population: Patients aged 60 years and older with diabetes type 2 from primary care, enlisted with the diabetes service of the Diagnostic Center in Etten-Leur (SHL)

All participants will be investigated at the cardiology out-patient department of the regional hospital (Oosterschelde Hospital in Goes, Zeeland, the Netherlands) during a single 1.5 hour standardised diagnostic assessment, including history taking, physical examination, electrocardiography, echocardiography, blood tests, and Health status questionnaires. Patients will be asked if we can contact them afterwards for follow-up and for repeating the questionnaires after three and 12 months.

Main study parameters/endpoints: Prevalence (with exact 95% confidence intervals) of (previously unrecognised) heart failure (systolic and 'isolated' diastolic) and the diagnostic value of signs and symptoms, NT-proBNP, electrocardiography and a combination of these items. The cost-effectiveness of different diagnostic strategies. Impact of heart failure and the combination of a new diagnosis with accordingly treatment on health status.

**Trial registration:**

CCMO register NL2271704108

## Background

Cardiovascular diseases account for up to 80% of the excess mortality in patients with diabetes type 2 (DM2) [1]. Processes underlying this excess cardiovascular risk include coronary atherosclerosis, microvascular disease and autonomic neuropathy [1]. Importantly, morphological myocardial abnormalities and eventually diabetic cardiomyopathy and heart failure (HF), i.e. left ventricular dysfunction with symptoms of heart failure play a role [2,3]. Notwithstanding the high rate of cardiovascular diseases and the availability of morbidity and mortality reducing treatment for heart failure, [4] up to now, only a few studies assessed the prevalence of HF in diabetic patients. These studies showed a prevalence of HF of 12-22% in the subgroup of diabetic patients who had been admitted to the hospital [5,6]. In another study, the incidence rate of HF in DM2 patients was about 2.5 times higher than in non-diabetic control subjects [7]. Importantly, the diagnosis of HF in the aforementioned studies was based on hospital discharge diagnoses. To our knowledge, there are no studies that assessed HF using a diagnostic strategy including echocardiography in a representative sample of patients with diabetes in primary care (not selectively in those admitted to a hospital) [8,9]. Hence, valid prevalence estimates of HF in patients with diabetes type 2 are lacking. Such studies are needed because the majority of patients with diabetes is managed in primary care [10]. The diagnosis of HF however is notoriously difficult in the early phases of the syndrome and in the presence of co morbidities, such as chronic obstructive pulmonary disease (COPD) and obesity [11]. Moreover, echocardiography, the cornerstone investigation to diagnose HF, [4] is usually not ready at hand for primary care patients. Studies indicate that 50% of all patients (thus not only those with DM2) with HF in primary care remain undetected [10]. Recently, our group showed that unrecognised HF was common (prevalence 20.5%) among primary care patients with COPD aged ≥ 65 years [12]. High prevalence of unrecognised HF can therefore also be expected in patients with diabetes in primary care.

We hypothesize that the prevalence of unknown heart failure in diabetic patients aged 60 years and over is relatively high (15% or more). Our study will provide valid and precise prevalence estimates of previously unrecognised HF in DM2 patients (relevant for secondary prevention). Comparison of different combinations of diagnostic tests to recognise HF will reveal the most cost-effective strategies to detect HF in patients with DM2. The strategy is expected to include some signs and symptoms (such as dyspnoea, orthopnoea, pulmonary crepitations, laterally displaced apical beat), B-type natriuretic peptide measurements (NT-proBNP)), and possibly electrocardiography. In a subset of patients straightforward echocardiography may prove to be cost-effective.

With information from our study the detection of previously unknown heart failure in diabetes patients can be improved, enabling the physician to initiate beneficial morbidity and mortality reducing heart failure treatment more timely.

### Primary objectives

- To assess the prevalence of (previously unrecognised) HF in primary care patients with diabetes type 2 (DM 2).

- To establish the most cost-effective diagnostic strategy to detect unrecognised HF in these patients.

### Secondary objectives

- To assess the impact of heart failure, and the combination of new diagnosis of HF with accordingly treatment in patients with diabetes type 2 on health status.

## Methods/design

### Study design

**Figure 1:**
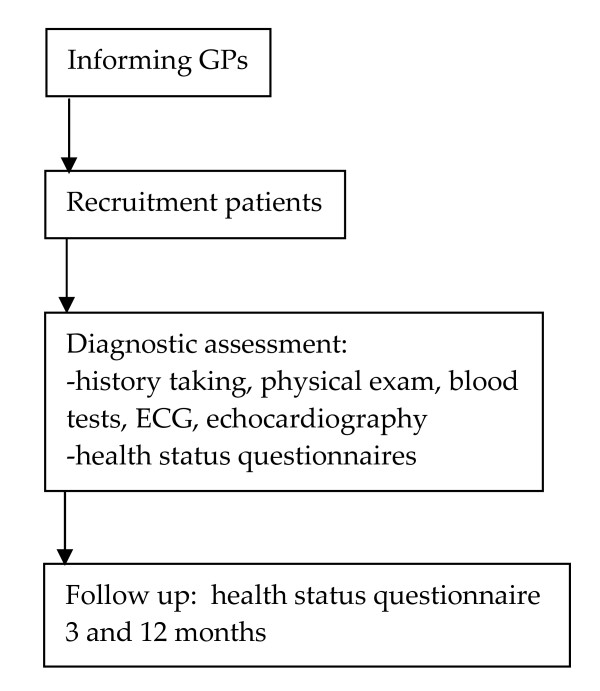
**Study scheme**.

A prospective diagnostic efficiency study. See figure 1 for the study scheme and table 1 for the measurement scheme.

**Table 1:** Measurement Scheme

		baseline	3 months	12 months
VISIT		1		
**Informed Consent**		X		

**Diagnostic assessment**				

History taking		X		

Physical examination		X		

ECG		X		

NT-proBNP		X		

Other laboratory assessments (HbA1C, Fasting Glucose, plasma creatinin, ureum; hsCRP, Hb, Ht)		X		

Echo		X		

**Questionnaire patient**	number of items			

Demographic parameters		X		

Diabetes Health Profile	32	X	X	X

SF36	36	X	X	X

EQ5D	06	X	X	X

Medication use		X	X	X

**Diagnostic centre and GP registration**				

Medical history		X		

Medical costs		X		

### Study population

Patients aged 60 years and over with diabetes type 2, enlisted with the diabetes service of the Center for Diagnostic Support in Primary Care (SHL), Etten-Leur are eligible. This is a representative sample of all patients with diabetes type 2 registered with a general practitioner. The standard care of the diabetes service consists of periodically serum glucose and HbA1c assessment and yearly monitoring of other laboratory parameters and fundoscopy, to help the general practitioners with the management of patients with diabetes. Furthermore, the SHL provides a supporting service to the general practitioners of diabetic nurses, who work according to the current diabetes guidelines.

In total 561 general practitioners in the region make use of the services of the Diabetic service of the SHL, with 48,175 patients with type 2 diabetes enlisted in the SHL service. In total, 100 nurse practitioners from the SHL support more than 200 general practitioners (GPs) in their work for diabetic patients. A random sample of approximately 1200 patients enlisted within the SHL diabetic service database and living within 60 kilometres of the cardiology outpatient department of the Oosterschelde hospital in Goes will be asked to participate in the study. To prevent duplicate investigations, patients known with a cardiologist-confirmed diagnosis of heart failure, will only be asked to fill out the questionnaires. They also will be asked for permission to scrutinize their medical files for co-morbidities and date of diagnosis of heart failure. The expected prevalence rate of already known HF is less than 5% (30 of the 600 responders).

### Power calculation

When the diagnostic value of several diagnostic variables or tests together needs to be quantified, no straightforward methods to estimate the required sample size are available. A 'rule of thumb' for each diagnostic determinant included in the analysis is at least 10 events in the smallest category (in our study those with heart failure) is recommended. Our study will include at the most 9 diagnostic determinants (e.g. items from history and physical examination summarised in a score, ECG, and NT-proBNP). Thus, 90 patients with previously unknown heart failure would be required. With an estimated prevalence rate of 15% of previously unknown heart failure, about 600 diabetic type 2 patients should participate. Assuming, based on previous experiences, a response rate of at least 50%, approximately 1200 patients will be invited.

### Patient invitation

Eligible patients receive an information letter with information about the study, and they are asked to send their answer back. If the patient is interested in the study, the patient receives detailed information about the study. Patients who are not willing to take part in the study will be asked for the reasons for not participating.

### Patient consent

Patients that are invited to participate in this study are entitled to choose whether or not to take part. Their decision will be voluntary and they should be competent to understand what the study involves. The information provided will be in the patient's own language.

### Documentation of consent

Written informed consent is obtained before any study procedure will be undertaken. The written consent form will be signed and dated by each participant and the researcher.

### Diagnostic assessment

All participants will be investigated at the cardiology out-patient department of the regional hospital (Oosterschelde Hospital in Goes, the Netherlands) during a single 1.5 hour standardised diagnostic assessment. The diagnostic measurements include: history taking (e.g. orthopnoea), physical examination (e.g. pulmonary crepitations, laterally displaced apical beat), electrocardiography, and echocardiography. Plasma B-type natriuretic peptide measurements, glucose levels, creatinin levels, and HbA1C will be measured a few days later at a local 'medical checkpoint' in the neighbourhood of the participant, during their regular blood sample taking for the diabetes service. Signs and symptoms will be assessed by a trained physician in a standardised manner. The clinical findings will be combined in a 'clinical score' [13]. Present medication use is asked for and will be checked, patients will be asked to take their medication to the outpatient department. Medical history and time since diagnosis of diabetes with all diabetes related and not-related (e.g. NSAIDs) medication will be checked in the electronic medical files of the general practitioners (GPs). Blood samples (20 ml) will be taken and after centrifugation specimens of plasma and cells will be stored at -70 degrees Celsius. B-type natriuretic peptides (BNP and NTproBNP) are released predominantly from the ventricular myocardium in response to myocardial stretch, such as in heart failure [14]. The plasma level of both BNP and the release split product amino-terminal proBNP (NT-proBNP) are closely related to left ventricular function [15]. We will measure NT-proBNP levels from plasma using a non-competitive immunoradiometric assay (Roche Inc., Germany).

A standard 12-lead electrocardiogram (ECG) will be recorded and classified according to the Minnesota coding criteria by an experienced and trained cardiologist. Echocardiography will be performed with a General Electric, Vivid 7 imaging system by an experienced cardiac sonographer. All echocardiographic images will be recorded and interpreted by a cardiologist, who is blinded to clinical data. Parameters from M-mode and two-dimensional echocardiography with Doppler analysis will be applied. The left ventricular ejection fraction (LVEF) will be assessed quantitatively or semi-quantitatively, when necessary. Diastolic function will be assessed by an integrated combination of Doppler measurements of the mitral inflow and Doppler tissue imaging (DTI) of the mitral annulus [16]. Inclusion of DTI creates the possibility to measure left ventricular relaxation and filling pressures load independently in a reproducible and feasible way [16-23].

### Reference standard of heart failure

Presence or absence of HF will be determined by an outcome panel consisting of two cardiologists and one GP. In analogy with earlier studies, the panel will use all available information from the diagnostic work-up, including echocardiography, but except the NT-proBNP results (to prevent incorporation bias pertaining to this particular test). Consensus diagnosis by an outcome panel is an established method in case an irreproachable reference standard is lacking, as is the case for HF [8,9]. Outcome panels have been successfully applied in earlier studies of HF by our group and the reproducibility of this method is high [13]. In case of no consensus the majority decision will be used. Patients classified as HF by the panel will be further classified as systolic or 'isolated' diastolic heart failure or 'isolated' right sided heart failure. For systolic heart failure, patients have to have an echocardiographic left ventricular ejection fraction ≤ 45% in combination with presence of symptoms indicative of heart failure (that is, orthopnoea, paroxysmal nocturnal dyspnoea, fatigue, peripheral oedema, nocturia more than twice a night, or any combination of these symptoms). For isolated diastolic ventricular dysfunction, patients have to have echocardiographic diastolic dysfunction and a left ventricular ejection fraction > 45%. For isolated diastolic heart failure patients have to have echocardiographic diastolic abnormalities in combination with indicative symptoms and signs (that is, peripheral or pulmonary fluid retention or raised jugular venous pressure) of heart failure [24] or indicative symptoms and echocardiographic left ventricular hypertrophy, atrial fibrillation, or anginal complaints [25].

### Assessment of diabetes

All patients registrated in the diabetic service of SHL have been diagnosed with diabetes according to the Diabetes Guidelines. The diagnosis diabetes was established if on two different days the fasting glucose level was above cut-off levels (6.0 mmol/l in capillary blood or 6.9 mmol/l in venous plasma or non-fasting glucose level > 11.0 mmol/l) or one glucose level > 11.0 mmol/l in combination with symptoms of hyperglycaemia, such as itching, thirst etc.

### Health status measurement

To assess the impact of (detecting and treating previously unknown) heart failure on the health status the participants will receive a questionnaire (including questions on health status) to be completed during their visit to the diagnostic centre at baseline. The group known with a cardiologist-confirmed diagnosis of heart failure will be visited at home. After 3 months and after 12 months from baseline measurement the participants will receive the same questionnaires, including an additional questionnaire with questions about possible change in medication.

In order to calculate quality-adjusted life years (QALYs) preference-based utilities will be measured with the EuroQol-5D instrument [26]. Generic health status will be assessed with the Short Form 36 (SF36) [27-29]. For disease specific health status the diabetes health profile (DHP) will be used [30]. These questionnaires are all widely tested and used.

### Cost-effectiveness analysis

A cost-effectiveness analysis of different strategies to detect unrecognized HF in patients with type 2 diabetes will be performed based on a social perspective according to international and national guidelines [31]. As a result of the primary data-analysis, various diagnostic strategies will be identified which differ in terms of the tests that are performed, the order of the tests, and the cut-off points used. Each strategy and each cut-off-point leads to different numbers of patients who are diagnosed as true positive (TP), false positive (FP), true negative (TN) and false negative (FN). To estimate the costs associated with these categories (FP, TP, FN, TN) a previously used and validated Markov model will be applied. The various strategies will be ranked by increasing cost-effectiveness ratios; the most optimal strategy in terms of cost effectiveness will be defined. It is important to note that there may be discrepancies in the most effective diagnostic strategy and the most cost-effective diagnostic strategy. The incremental cost-effectiveness of those tests that add to the best available diagnostic strategy, compared to the sub-optimal diagnostic strategy is of importance, in other words the additional value of the more expensive and most discriminating tests. For measuring direct costs, resource quantities are collected from the case record forms, and prices will be based on market prices or tariffs for the investigations performed in the study. Only relevant indirect medical costs are taken into consideration such as patient time and travel costs. When comparing diagnostic strategies, however, indirect costs are less relevant because all investigations can be performed in a single setting at the outpatient department, irrespective of the number of investigations. Both absolute costs and incremental costs of different diagnostic strategies will be taken in consideration.

A sensitivity-analysis will be performed in order to estimate the susceptibility of cost-effectiveness ratio's to variation in prior assumptions and choices, including a 'worst case - best case' comparison.

### Statistical analysis

Prevalence of (previously unrecognised) heart failure (systolic and 'isolated' diastolic) will be calculated with exact 95% confidence intervals. The crude association of each diagnostic test (including the 'clinical score') with the presence or absence of HF will first be quantified by calculating predictive values, sensitivity, specificity and likelihood ratios by using univariate logistic regression analysis. Those with a p-value < 0.15 in the univariate analysis will subsequently be included in a multivariate logistic regression analyses to determine their independent contribution to the diagnosis of HF. In the analyses the chronology in which investigations are performed in practice will be followed. First of all, the diagnostic value of the signs and symptoms score will be assessed. Then NT-proBNP and ECG will be added, first separately and then in different combinations, to quantify their added diagnostic value, using the likelihood ratio test at a p-value of < 0.10 [32]. Areas under the ROC curve will be calculated for different combinations of the parameters to further quantify the diagnostic accuracy. Echocardiographic variables will not be evaluated separately on their diagnostic value because they would likely receive an overriding weight in the consensus judgement (i.e. diagnostic outcome assessment) [33]. In the cost-effective analysis, however, a separate diagnostic strategy to detect previously unrecognised HF by means of echocardiography only, will be evaluated, based on the echocardiographic findings. To assess the impact of heart failure, and diagnosis and treatment of heart failure in diabetes type 2 patients on health status, the scores on the Health status questionnaires of the three groups (no heart failure, known heart failure and previously unknown heart failure) will be compared at baseline (t0) and on 3 months and 12 months. Also the difference between 0-3 months, 3-12 months and 0-12 months within patients, will be compared between the groups (T-test and ANOVA).

### Regulatory authority approval

This study will be prepared, conducted and reported in compliance with the Dutch Law on Medical research with humans (WMO). The study will be conducted according to the current version of the Declaration of Helsinki.

### Ethics committee approval

The Medical Ethical Committee of the University Medical Center Utrecht (METC) assessed and approved the protocol, the patient consent forms, the patient information letters, and the letter to the General Practitioner.

## Abbreviations

CEA: Cost-Effectiveness Analysis; HF: (Congestive) heart failure; DM: Diabetes Mellitus; ECG: Electrocardiography; Echo: Echocardiography; GP: General Practitioner; NT-pro-BNP: Amino-terminal pro-B-type natriuretic peptide; SHL: Center for diagnostic Support in Primary Care (Stichting Huisartsen Laboratorium)

## Competing interests

The authors declare that they have no competing interests.

## Authors' contributions

LB participated in the design and coordination of the study, drafted the manuscript. FH participated in the design of the study, helped to draft the manuscript and is a member of the panel. MC revised the manuscript and is a member of the panel. AL participated in the design and coordination of the study. ML is a member of the panel. HS and AW participated in the design of the study. GR revised the manuscript. PH participated in the coordination of the study. AH is responsible for conception of the study, participated in the design and revising the manuscript. All authors read and approved the final manuscript.

## Pre-publication history

The pre-publication history for this paper can be accessed here:

http://www.biomedcentral.com/1471-2458/9/479/prepub

## References

[B1] Blendea MC McFarlane SI Isenovic ER Gick G Sowers JR Heart disease in diabetic patients Curr Diab Rep 2003 3 223 9 10.1007/s11892-003-0068-z 12762970

[B2] Giles TD The patient with diabetes mellitus and heart failure: at-risk issues Am J Med 2003 8 115 Suppl 8A 107S 110S 10.1016/j.amjmed.2003.09.017 14678875

[B3] Piccini JP Klein L Gheorghiade M Bonow RO New insights onto diastolic heart failure: role of diabetes mellitus Am J Med 2004 116 Suppl 5A 64S 75S 10.1016/j.amjmed.2003.10.021 15019864

[B4] Swedberg K Cleland J Dargie H Drexler H Follath F Komajda M Tavazzi L Smiseth OA Gavazzi A Havrich A Hoes A Guidelines for the diagnosis and treatment of chronic heart failure: executive summary (update 2005): The task force for the diagnosis and treatment of chronic heart failure of the European Society of Cardiology Eur Heart J 2005 26 1115 40 10.1093/eurheartj/ehi166 15901669

[B5] Bertoni AG Hundley WG Massing MW Bonds DE Burke GL Goff DC Heart failure prevalence, incidence, and mortality in the elderly with diabetes Diabetes Care 2004 26 699 703 10.2337/diacare.27.3.699 14988288

[B6] Nichols GA Hillier TA Erbey JR Brown JB Congestive heart failure in type 2 diabetes. Prevalence, incidence, and risk factors Diabetes Care 2001 24 1614 19 10.2337/diacare.24.9.1614 11522708

[B7] Nichols GA Gullion CM Koro CE Ephross SA Brown JB The incidence of congestive heart failure in type 2 diabetes Diabetes Care 2004 27 1879 84 10.2337/diacare.27.8.1879 15277411

[B8] Moons KG Grobbee DE When should we remain blind and when should our eyes remain open in diagnostic studies? J Clin Epidemiol 2002 55 633 6 10.1016/S0895-4356(02)00408-0 12160909

[B9] Bossuyt PM Reitsma JB Bruns DE Gatsonis CA Glasziou PP Irwig LM The STARD statement for reporting studies of diagnostic accuracy: explanation and elaboration Clin Chem 2003 49 7 18 10.1373/49.1.7 12507954

[B10] Wheeldon NM MacDonald TM Flucker CJ McKendrick AD McDevitt DG Struthers AD Echocardiography in chronic heart failure in the community Q J Med 1993 86 17 23 8438044

[B11] Remes J Miettinen H Reunane A Pyorala K Validity of clinical diagnosis of heart failure in primary health care Eur Heart J 1991 12 315 21 2040313 10.1093/oxfordjournals.eurheartj.a059896

[B12] Rutten FH Cramer M-JM Grobbee DE Sachs APE Kirkels JH Lammers J-WJ Hoes AW Unrecognized heart failure in elderly patients with stable chronic obstructive pulmonary disease Eur Heart J 2005 26 1887 1894 10.1093/eurheartj/ehi291 15860516

[B13] Rutten FH Moons KGM Cramer M-JM Grobbee DE Zuithoff NPA Lammers J-WJ Hoes AW Recognising heart failure in elderly patients with stable chronic obstructive disease in primary care: a cross-sectional diagnostic study BMJ 2005 331 1379 76 10.1136/bmj.38664.661181.55 16321994 PMC1309648

[B14] Rutten FH Walma EP Kruizinga GI Bakx HCA Van Lieshout J NHG-standaard Hartfalen, eerste herziening Huisarts Wet 2005 48 64 76

[B15] Wright SP Doughty RN Pearl A Gamble GD Whalley GA Walsh HJ Plasma amino-terminal pro-brain natriuretic peptide and accuracy of heart failure diagnosis in primary care: a randomised controlled trial J Am Coll Cardiol 2003 42 1793 800 10.1016/j.jacc.2003.05.011 14642690

[B16] Schiller NB Shah PM Crawford M DeMaria A Devereux R Feigenbaum H Recommendations for quantitation of the left ventricle by two-dimensional echocardiography. American Society of Echocardiography Committee on Standards, Subcommittee on Quantitation of Two-Dimensional Echocardiograms J Am Soc Echocardiogr 1989 2 358 67 2698218 10.1016/s0894-7317(89)80014-8

[B17] Folland ED Parisi AF Moynihan PF Jones DR Feldman CL Tow DE Assessment of left ventricular ejection fraction and volumes by real-time, two-dimensional echocardiography. A comparison of cineangiographic and radionuclide techniques Circulation 1979 60 760 66 476879 10.1161/01.cir.60.4.760

[B18] Willenheimer RB Israelsson BA Cline CM Erhardt LR Simplified echocardiography in the diagnosis of heart failure Scand Cardiovasc J 1997 31 9 16 10.3109/14017439709058063 9171143

[B19] Tsang TS Barnes ME Gersh BJ Bailey KR Seward JB Left atrial volume as a morphophysiological expression of left ventricular diastolic dysfunction and relation to cardiovascular burden Am J Cardiol 2002 90 1284 89 10.1016/S0002-9149(02)02864-3 12480035

[B20] Garcia MJ Thomas JD Klein AL New Doppler echocardiographic applications for the study of diastolic function J Am Coll Cardiol 1998 32 865 75 10.1016/S0735-1097(98)00345-3 9768704

[B21] Oh JK Appleton CP Hatle LK Nishimura RA Seward JB Tajik AJ The noninvasive assessment of left ventricular diastolic function with two-dimensional and Doppler echocardiography J Am Soc Echocardiogr 1997 10 246 70 10.1016/S0894-7317(97)70062-2 9109691

[B22] Schena M Clini E Errera D Quadri A Echo-Doppler evaluation of left ventricular impairment in chronic cor pulmonale Chest 1996 109 1446 51 10.1378/chest.109.6.1446 8769491

[B23] Bursi F Weston SA Redfield MM Systolic and diastolic heart failure in the community JAMA 2006 296 2209 16 10.1001/jama.296.18.2209 17090767

[B24] Zile MR Heart failure with preserved ejection fraction: is this diastolic heart failure? J Am Coll Cardiol 2003 41 1519 22 10.1016/S0735-1097(03)00186-4 12742292

[B25] Vasan RS Benjamin EJ Levy D Congestive heart failure with normal left ventricular systolic function. Clinical approaches to the diagnosis and treatment of diastolic heart failure Arch Intern Med 1996 156 146 57 10.1001/archinte.156.2.146 8546548

[B26] Brooks R Rabin R de Charro F The Measurement and Valuation of Health Status Using EQ-5D: A European perspective 2002 Kluwer Academic Publishers

[B27] Ware JE Snow KK Kosinski M Gandek B SF-36 Health Survey Manual and Interpretation Guide 1993 Boston, MA: New England Medical Center, The Health Institute

[B28] Aaronson NK Muller M Cohen PD Translation, validation, and norming of the Dutch language version of the SF-36 health survey in community and chronic disease populations J Clin Epidemiol 1998 51 1055 68 10.1016/S0895-4356(98)00097-3 9817123

[B29] Ware JE Jr Kosinski M Bayliss MS Comparison of methods for the scoring and statistical analysis of SF-36 health profile and summary measures: summary of results from the Medical Outcomes Study Med Care 1995 33 4 Suppl 264 79 7723455

[B30] Meadows K Steen N McColl E Eccles M Shiels C Hewison J Hutchinson A The Diabetes Health Profile (DHP): a new instrument for assessing the psychosocial profile of insulin requiring patients--development and psychometric evaluation Qual Life Res 1996 5 2 242 54 10.1007/BF00434746 8998493

[B31] Oostenbrink JB Koopmanschap MA Rutten FF Standardisation of costs: the Dutch Manual for Costing in economic evaluations. Pharmacoeconomics 2002 20 7 443 54 12093300 10.2165/00019053-200220070-00002

[B32] Harrell FE Lee KL Mark DB Multivariable prognostic model issues in developing models evaluating assumptions and adequacy, and measuring and reducing errors Stat Med 1996 15 361 87 10.1002/(SICI)1097-0258(19960229)15:4<361::AID-SIM168>3.0.CO;2-4 8668867

[B33] Moons KG Biesheuvel CJ Grobbee DE Test research versus diagnostic research Clin Chem 2004 50 473 6 10.1373/clinchem.2003.024752 14981027

